# Reply: Lead-preserving Strategies for Pacemaker Pocket Infection: Who, When and How?

**DOI:** 10.1016/s0972-6292(16)30571-x

**Published:** 2012-12-02

**Authors:** Adam J Nelson, Rishi Puri, Peter J Psaltis, Prashanthan Sanders, Glenn D Young

**Affiliations:** 1 Cardiovascular Research Centre, Royal Adelaide Hospital; 2 Cardiovascular Investigation Unit, Royal Adelaide Hospital

**Keywords:** Pacemaker, infection, conservative treatment

We would like to thank Drs. Shafiee and Kazemisaeed for their comments [1] with reference to our case "Povidone Iodine Irrigation - A possible alternative to lead extraction" [2]. Indeed we read their case [3] with keen interest, along with the experience ofothers who have similarly documented treatment of pacemaker/device pocket infection with a variety of lead-preserving techniques, which mostly encompass systemic antimicrobials with a variable period of antiseptic solution irrigation of the infected pocket. We support Drs Shafiee and Kazemisaeed's comments and build on their recommendations.

1. Lead extraction. In keeping with standard surgical convention, the standard of care suggests all patients with infected pockets undergo full hardware extraction in order to reduce their risk of recurrent infection [4,5]. Removal of all hardware, including leads which may have been present for years, is not without difficulty and risk. Novel methods of lead extraction (laser or radiofrequency powered extraction sheaths) have resulted in published success rates in excess of 95% [6], however the risk of potential complications (myocardial avulsion or vascular tear with subsequent tamponade, air embolism, septic shock and death) is not insignificant. As previously discussed these complication risks are largely dependent upon operator experience, and the availability of cardiac surgical back up. As such, this expertise is limited to large volume centres with appropriate on site facilities. Where this is not possible, a lead-preserving strategy could be considered.

2. Patient selection. Highest success rates have been observed with strict inclusion/exclusion criteria for embarking upon a lead-preserving strategy; most importantly the absence of bacteraemia or signs of endocarditis [7]. Given the inherent risk of recurrent infection and endocarditis, we would recommend careful selection and informed consent of patients for lead-preserving strategies, and that they be offered only where hardware removal is not possible or plausible. This could include but not be limited to:

- unextractable leads (up to 20% inspite of new techniques [8])

- frail patients unable to tolerate cardiac surgery

- resource limited areas without cardiac surgery facilities

- patient unwillingness to proceed with lead extraction (our case report)

3. Technique and irrigation solution. With only a handful of case reports and case series in the current literature, it is impossible to make definitive recommendations about the appropriate irrigation technique, time frame and choice of antiseptic solution. We reiterate sentiment from Drs. Shafiee and Kazemisaeed with respect to the need for randomised data on the efficacy of each technique. However with small numbers of documented cases, a variety of reported (and variably incubated) pathogens, heterogeneity in patient profile, and increasing competition with the growing interest in lead-extraction techniques and availability of cardiac surgical support, this important clinical question may remain unresolved. A detailed review of antiseptic solutions is outside the scope of this commentary, however considerable debate exists surrounding the efficacy of antiseptic solutions for pre-procedural skin preparation through to irrigation of established wounds. Evidence from the intensive care environment suggest chlorhexidine to be a superior antiseptic to povidone-iodine in preventing infection of central venous catheters (CVCs) [9],although its performance in animal models of established wound therapy has been mixed. Some data suggest inhibition of wound healing [10], potentially from toxicity [11], whereas others report acceleration of the healing response [12,13]. With human data lacking and the heterogeneity of its application in the present literature, the 'ideal' antiseptic remains unknown, particularly in the unique setting of a prosthesis or implant.

We applaud the IPEJ for the opportunity to continue this dialogue on what remains an important therapeutic alternative for treatment of pacemaker pocket infection.
